# A Grounded Theory Approach to Navigating Infertility Care During U.S. Military Service

**DOI:** 10.1093/milmed/usac174

**Published:** 2022-06-30

**Authors:** Jennifer Buechel, Carmen N Spalding, Whitney W Brock, Judy L Dye, Natalie Todd, Candy Wilson, Eileen K Fry-Bowers

**Affiliations:** Naval Submarine Medical Research Laboratory, Groton, CT 06349, USA; Bioskills Simulation Training Center, Navy Medicine Readiness and Training Command San Diego, San Diego, CA 92134, USA; Department of Pediatrics, Neonatal Intensive Care Unit, Kaiser Permanente San Diego Medical Center, San Diego, CA 92123, USA; University of California San Diego Health, La Jolla, CA 92037, USA; Rady Children’s Hospital San Diego, San Diego, CA 92123, USA; San Diego State University School of Nursing, San Diego, CA 92182, USA; The Geneva Foundation, Tacoma, WA 98402, USA; Daniel K. Inouye Graduate School of Nursing, Uniformed Services University of the Health Sciences, Bethesda, MD 20814, USA; Hahn School of Nursing and Health Science, University of San Diego, San Diego, CA 92110, USA

## Abstract

**Introduction:**

In this study, we aimed to understand how active duty service members and their partners navigate the infertility care process within the Military Health System (MHS) while managing a military career.

**Materials and Methods:**

We obtained Institutional Review Board approval to employ a qualitative design using grounded theory methods. We recruited participants using purposive sampling, followed by theoretical sampling. We derived data from demographic questionnaires and semi-structured interviews. Consistent with grounded theory methods, we began analysis with line-by-line coding and moved to focused coding. We employed constant comparative analysis throughout the process to name, categorize, and conceptualize data and relationships.

**Results:**

The participants included 28 patients, five partners, nine health care providers, and two military leaders. The infertility care process began with active duty service members and their partners recognizing the desire to have a child and discovering infertility, followed by deciding to seek infertility care. The experience was temporally bound within the context of the military environment. We identified the following themes, which described facilitators and barriers to accessing care: Duty station location, career stage, military versus the civilian cost of services, command climate, and policy. These facilitators and barriers varied widely across the Department of Defense (DoD), which resulted in fragmented and inconsistent care cycles, contributed to emotional and physical stress, and created tension between career progression and family formation.

**Conclusions:**

Understanding how military couples perceive and manage demands of infertility care may enhance access to care, decrease patient costs, improve outcomes, result in better support for military couples who experience infertility, and ultimately improve the health and military readiness of our armed forces. The results support the need for action by providers, policy makers, and military leaders to develop effective infertility treatment programs and policies in the DoD.

## INTRODUCTION

In the United States, one in every six couples of childbearing age experiences infertility, defined as inability to achieve a clinical pregnancy after 12 months of unprotected sexual intercourse.^1–3^ The overall incidence of infertility for U.S. active duty service members (ADSMs) is 32.3 cases per 10,000 person-years for males and 79.3 cases per 10,000 person-years for females.^4,5^

**FIGURE 1.**
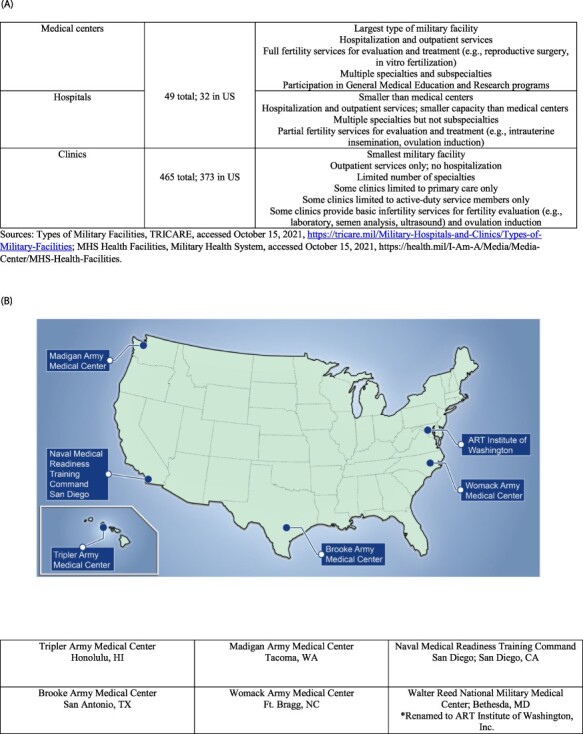
Reproductive endocrinology services available to military service members and their partners. (A) Types of military treatment facilities offering infertility services. Sources: Types of Military Facilities, TRICARE, accessed October 15, 2021, https://tricare.mil/Military-Hospitals-and-Clinics/Types-of-Military-Facilities; MHS Health Facilities, Military Health System, accessed October 15, 2021,https://health.mil/I-Am-A/Media/Media-Center/MHS-Health-Facilities. (B) Map of military treatment facilities offering assisted reproductive therapy services.

Assisted reproductive therapy (ART), including fertility medication, intrauterine insemination (IUI), in vitro fertilization (IVF), egg and sperm cryopreservation, and surrogacy, is an option for couples experiencing infertility. Although ART improves chances of conception, ART procedures require rigorous medical evaluations, invasive procedures, and lengthy treatment cycles. Although ART is available to ADSMs and their families at six military treatment facilities (MTFs), access varies (Fig. 1) and is not fully covered by TRICARE insurance.^6^

Military service presents unique challenges to reproduction, especially to infertility treatments. Among ADSMs, permanent change of station (PCS), temporary duty, and deployment may disrupt, delay, or limit access to ART. Military leaders’ and health care providers’ support for ART may influence ADSMs’ and their partners’ decision-making processes and experiences. Moreover, the infertility care experience can cause emotional and physical crises for ADSMs and their partners.

Despite the potential impacts on mission capability, few studies address the unique infertility care experiences of this population. Our qualitative study seeks to understand how ADSMs and their partners navigate the ART process within the Military Health System (MHS) while managing a military career.

## METHODS

Data collection and analysis were informed by a constructivist grounded theory approach.^7^ This approach focuses on generating theories for complex social processes through inductive analysis of the data gathered rather than preexisting theoretical frameworks. After written consent, participants completed a demographic survey and participated in a semi-structured interview. In-person interviews were conducted at an MTF that provides ART for approximately 150 IVF cycles, treats over 600 new patients, and offers 6,000 appointments annually. Phone interviews were also conducted with participants within the United States and overseas. We asked questions about infertility and participants’ perceptions, implications, and decision-making processes regarding ART while serving in the military. Field notes and memos augmented the qualitative data. We used constant comparative analysis until reaching theoretical saturation and identified core categories for a proposed theory. Rigor and trustworthiness were assured through peer review of analysis and auditing to check for biases. Demographic data analysis was performed using Statistical Package for the Social Sciences; qualitative data were managed using NVivo (QSR International). The local Institutional Review Board approved the study before launch.

## RESULTS

### Participant Selection

We purposively selected participants to provide perspectives on their infertility journey within the MHS while managing a military career. Participants included ADSMs, reservists, veterans, significant others, military leaders, and health care providers caring for infertile patients. A total of 135 people expressed interest in the study, and 38 consented and were interviewed. These participants included 28 ART patients, 5 partners, 9 health care providers, and 2 military leaders (some participants were interviewed under multiple categories; see Table I). The experiences of the health care providers substantiated the ART patients’ and partners’ perceptions.

**TABLE I.** Background of Patients and Partners Receiving Assisted Reproductive Therapy, *N* = 32^a^

Variable	Frequency, *n* (%)^b^
Gender^c^	
Male	5 (15.6)
Female	27 (84.4)
Age (years)	*M* = 36.125
25-30	4 (12.5)
31-35	14 (43.8)
36-40	6 (18.8)
41-45	4 (12.5)
46-50	4 (12.5)
Race/ethnicity^d^	
White	25 (78.1)
Black	2 (6.25)
Hispanic	1 (3.13)
Asian	2 (6.25)
Other	2 (6.25)
Education completed	
Some college	3 (9.38)
Bachelor’s degree	10 (31.3)
Master’s degree	14 (43.8)
Doctorate	5 (15.6)
Relationship status	
Single	2 (6.25)
Casually dating	1 (3.13)
Seriously dating	1 (3.13)
Married	28 (87.5)
Military status	
Active duty	22 (68.8)
Reservist	2 (6.25)
Retiree	1 (3.13)
Dependent spouse	7 (21.9)
Branch of the military	
Navy	17 (53.1)
Army	5 (15.6)
Coast Guard	3 (9.38)
N/A	7 (21.9)
Rank/pay grade	
Enlisted	5 (15.6)
Officer	20 (62.5)
N/A	7 (21.9)
Years in the military	*M* = 13.08
1-5	3 (9.38)
6-10	9 (28.1)
11-15	6 (18.8)
16-20	3 (9.38)
21-25	2 (6.25)
26-30	2 (6.25)

a One participant did not complete the online demographic survey.

b Percentages may not add up to 100 because of rounding.

c Participants were asked to choose between two options for sex: Male or female.

d Participants were asked to identify their race or ethnicity.

Inclusion criteria: (1) active duty service members, reserves, veterans, or TRICARE beneficiaries currently experiencing or who have experienced infertility in the past 5 years; (2) spouses, significant others, or surrogates of active duty service members, reserves, veterans, or TRICARE beneficiaries who currently experience or who have experienced infertility in the past 5 years; (3) 18 years of age or older; and (4) speak English.

Exclusion criteria: (1) Minors; (2) participants who are unable to provide consent; (3) participants who are cognitively impaired; and (4) participants who are unable to verbally communicate in English.

### Theoretical Framework

**FIGURE 2.**
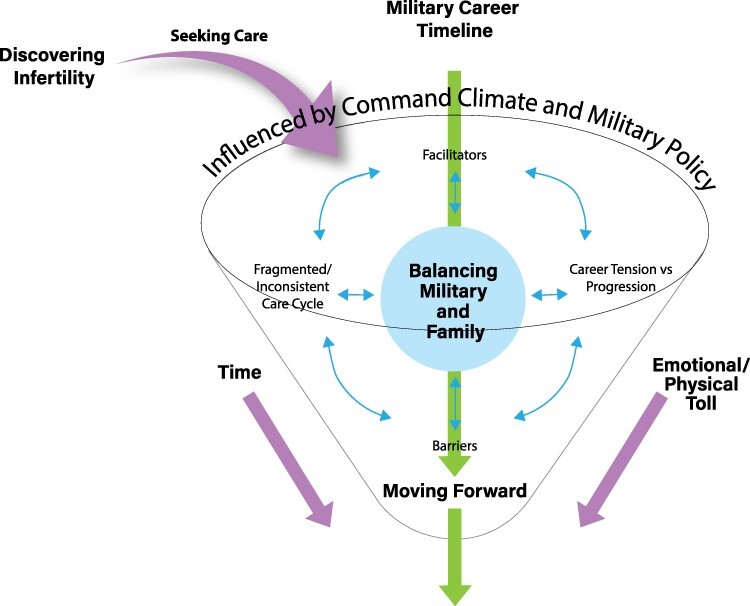
Navigating infertility in military service.

**FIGURE 3.**
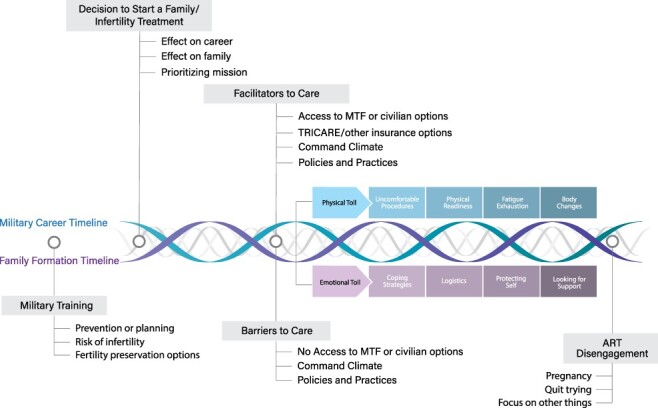
Time and contextually bound infertility process.

The infertility experience of ADSMs and their partners is temporally bound and occurs within the context of military culture and careers. The experience begins with recognizing the desire to have a child, discovering infertility, then deciding to seek ART. Biological limitations and career expectations require the ADSM, with or without their partner, to negotiate tensions between career progression and family formation. This tension takes a physical and emotional toll on those involved (Figs. 2 and 3).

#### Time

ADSMs’ desire for family formation is a time-bound process complicated by their military career and expectations for career progression. Frequently, a diagnosis of infertility is made while at least one partner is in the military. They then begin a complex journey of seeking care while navigating career progression amidst time-bound fertility considerations. The passage of time also influences decision-making processes and represents both obstacles and opportunities.

Once participants decided to engage in ART, seeking care required alignment with mission or career requirements.

“My husband got deployed and that reset everything. Then you have to start all over …”

“Army typically get assigned for 2 to 3 years at a duty station. But by the time you get care, by the time you’re done with the waitlist [for ART] … your 2 years are almost up.”

#### Military culture

The military represents a mission-driven structure with distinct subcultures. Military culture embodies selfless service and teamwork that embraces a warrior ethos. This self-sacrifice extended to ADSMs’ decision-making. ADSMs and their partners frequently prioritized the military mission, delaying ART and family formation. Dual military couples, who received ART only if their schedules aligned and they obtained leaders’ approval, were especially affected.

“It was a mission first kind of thing. And then we tried to not necessarily put it on the back burner, but, if the schedule allowed it, then we do it.”

ADSMs described family formation coming second to their duty obligation, sensing that leaders and colleagues would only approve time away if ART did not impact the mission.

“People, once they join the military, especially on the female side, are very much told about the risk [of] getting pregnant, and how you need to be responsible and think about the mission, and make sure you’re timing it and have a plan.”

“I got pregnant in December and cried and cried and cried about that because I thought I’d be in trouble’cause I now can’t deploy.”

### Facilitators and Barriers to Access and Receiving Care

ADSMs and their partners encountered numerous facilitators and barriers to accessing and receiving ART, particularly related to duty station, career stage, costs, command climate, policies, and personal tolls.

#### Duty station location

Duty station location, tour length, and PCS were principal facilitators or barriers to ART. Most duty station locations were based on billet assignments, occupational requirements, career pipelines, and timing.

##### Facilitators.

Participants stationed and/or living near infertility clinics expressed gratitude, ease of the ART process, and stability of the health care provider.

“I feel like it’s, honestly, with complete luck that I got in out here because I know quite a few friends of mine that are trying to get into Naval Center San Diego for infertility can’t.”

“We’ll probably try to come back [to] Madigan, which is easier and less expensive … I work here at Madigan, and going on the civilian route, sometimes I’ve got to drive either 40 minutes away or an hour away for labs or ultrasounds and things.”

##### Barriers.

Lack of access to specialists, lengthy travel distances, and limited availability of clinic services were barriers to seeking and receiving ART.

“We gotta stay near this clinic …’cause it’s extremely dangerous to transfer your embryos.”

“I was gonna have to be negotiating my orders. Most likely, thinking that they might be sending me overseas … where freezing my eggs, or fertility treatments if I end up in Guam, might not be an option.”

“Three-hour drive there and then another three-hour drive back. And in February and March I had to work a lotta half days and then leave and try to get the last appointment here.”

For dual active duty couples, duty station co-location and PCS orders presented challenges in coordinating care.

“We were finally ready to start the process, he had transferred. I stayed back for a year … so we lived apart for a year. And then, when it came time for me to transfer … he was in Charleston. I requested to go to Charleston, and they gave me orders to Georgia.”

#### Career stage

ADSMs distinguished between career stages as operational or deployable; nonoperational or non-deployable; and pipelines to leadership, promotion, school, or training. These stages were important for career progression and ART timing and access.

##### Facilitators.

Nonoperational/non-deployable and service/academic career stages were described as time periods that supported a focus on family. Participants, especially females, pursued ART during these times, believing them more manageable for balancing work and family.

“I took out my IUD, and was transitioning to a school environment where I was leaving sea duty, and I was allowed to get pregnant.”

“She’s doing school for 2 years, so it wouldn’t impact any mission if she were pregnant.”

##### Barriers.

Time spent in operational commands often required frequent travel, resulting in less time at home. During this career stage, participants experienced difficulty scheduling ART appointments and limited opportunities for timed ART cycles.

“I feel bad for women who are trying to get pregnant and their husband’s been deployed for 12 months. Or a woman who has to deploy, another 9-month window, 9 failed chances to get pregnant, and there comes a point where every month is precious.”

“The civilian sector women have the same challenges, but typically they don’t have to navigate deployment and shore duty … in the way that military members would.”

#### Service costs

Costs associated with ART can be excessive and influenced decisions to seek care, especially for ADSMs in junior ranks.

##### Facilitator.

Access to an MTF, where ART costs are reduced or covered by TRICARE, was a facilitator, according to ADSMs who used such services.

“I feel very fortunate to only spend $6,500 to have an opportunity to do IVF [at an MTF] because my cousin that lives in San Diego [civilian clinic] did hers and she spent up to $35,000.”

“IUIs at this [MTF] hospital are free, versus paying out of pocket. A lot of the places out in San Diego are quoting $1,200 just to do IUIs.”

##### Barrier.

Conversely, participants unable to access ART through an MTF were burdened by costs. Most insurance plans do not provide infertility care coverage.

“The procedure itself, which is $15,000 … $18,000 for the implantation and … fertilization … we saved up the money for what we need, but for most families, they’re not that lucky. … then you have to think about how much the interest rate is on that loan. How long are you gonna be paying that back?”

#### Command climate

The presence or absence of a supportive command climate appeared to be related to leaders’ ART knowledge and understanding, their own experiences, their empathy or apathy toward the situation, and perceived or actual gender differences. ADSMs sensed that leaders with personal experience with infertility and leaders with families were more supportive and empathetic.

##### Facilitators.

Participants believed positive or negative communication with leadership and the perception of supportive or unsupportive leaders were central to accessing and receiving ART. For example, supportive leaders were key to allowing time to access reproductive care.

“His chief … was actually a female who had had some infertility issues, so she understood … she was very flexible.”

Military community support and camaraderie played a role in participants’ perception of their command climate. Participants who felt part of a unique community or “their own little world” felt supported and cared for during their journey.

“… considering the community he was in … we probably got a lot of more hands-on treatment than he probably would’ve gotten if we had been on the boat … XX is very, very family oriented because they deploy so often … they literally have their own little world.”

##### Barriers.

Participants who viewed their leaders as unsupportive reported not receiving time off for appointments and those leaders prioritized mission needs over personal requests. Female officers expressed little support from leaders or colleagues regarding their decisions about infertility care.

“Army will never put our family ahead of what they’re doing … I think most of our leadership wouldn’t support what we were tryin’ to do if it wasn’t during school like what we’re trying now.”

Participants were hesitant to approach leaders and felt they would not be supported:

“I didn’t want to get this label, because the Coast Guard is so small that I just feel like if you get that reputation, [it’s] gonna be carried throughout your career.”

One participant described negative feelings in response to a perceived lack of support:

“I have a lotta anger and frustration at my command for not being supportive of this because I’ve been in 12 years. I’ve done two deployments. I’ve done numerous field problems, everything. I’ve gone everywhere the Army has wanted me to go without much of a fuss other than … when I was starting to get stationed at XX saying … this doesn’t have what I need. And the minute that I feel like I needed a little something then they don’t wanna be supportive.”

Female ADSMs expressed not feeling supported by their peers and hearing insulting comments that amplified gender inequity and lack of respect.

“It’s a very small command, so they have said things … my due date is November 9th, … with maternity leave, that gives me Thanksgiving, Christmas, and New Year’s Eve off … people have said comments to me like, ‘Oh, that was good timing … you planned that well.’ … If you only knew the process of everything we’ve been through just to have this child.”

#### Policy

##### Policy knowledge.

Not all participants or their leaders knew of or understood the policies that affected ART.

“I didn’t know about any of this stuff. There’s very little education out there, and the military doesn’t do a great job with getting that out to you.”

“I honestly don’t know what we currently do. I guess I would need more education on that if I were to have a staff member that were going through it.”

No one understands it [IVF or IUI]. … It’s very difficult being in the Army having to go through this process and not having your leadership support your process.”

Inconsistent understanding of policy led to inequities and disparities related to treatment, which in turn resulted in geographic variability regarding almost every aspect of receiving ART. Each ADSM had unique needs, and each local command followed its own policy practices.

“It seems to me … that not only with infertility issues, but … with the same-sex aspect, that either it’s policies in place … that just don’t make sense, for all circumstances, or a lack of like flexibility or just awareness.”

A non-partnered participant reported that a lack of clear understanding on policy regarding ART requirements resulted in them having to contest health care providers’ and leaders’ decisions.

“I could prove that I had 11 IUIs that didn’t work, 1 IVF that didn’t work. I had all the documentation from the doctor requesting that I have it, and they still said you can’t prove that you’re infertile or you’re needing infertility treatments … because you’re single.”

##### Gendered impacts.

Perceived gender inequities in military policy influenced ADSMs’, health care providers’, and leaders’ decisions and understanding of fertility and ART. Many female ADSMs expressed concern that if they chose to become pregnant with ART, they could be perceived as unable to perform their jobs, which would have negative consequences for their career.

“Unspoken rule that if you did get pregnant during operational, then you can’t do your job, so basically you’re, I don’t wanna say … blacklisted, but you’re gonna be taken outta your squadron, you’re gonna be moved somewhere else.”

“Men can have kids whenever and it’s no problem’cause they’re always ready and they don’t have to be there. With women, it takes you out of the fight for a very long time, and if you’re tryin’ to compete for jobs and everything, … And I know a few people who have had kids and have been fine, but it definitely is a huge setback when our male counterparts don’t have to do that.”

Participants suggested that greater attention to fertility by leaders is needed.

“At no time in my leadership development, or career counseling, or medical school [did] anyone say … you really gotta think about your age, and … preserving your fertility, and the implications of deployment on fertility … or long work hours on fertility …’ All of these things have an effect.”

“Instead of just having GMTs [General Military Training] talking about how to have safe sex, they should also say, ‘This is how your fertility drops after—’ I think that’s just as important … and what we’re doing to our female service members is a disservice if they’re not educated on that.”

##### Decisions on medical necessity.

Confusing terminology impacted the understanding of ART policies. Medical care, military readiness policies, and insurance delineations often conflicted on whether ART services were elective or medically necessary.

“With [my leadership] telling me at one point that it wasn’t medically necessary for me to come to my appointment after I had already began my injections a week in. And I’m like what do you want me to do with these heavy bloated ovaries at this point?”

One leader who underwent ART described ambivalence about subordinates who requested ART:

“I was kinda torn about it, especially now that I’m a leader.… I do think we need to offer sailors the opportunity to go through the treatment, but it’s elective in a way … I’m trying to do it on my own, but so is everyone else.”

When asked about policy development or reform, participants felt having clear definitions and uniform treatment options for accessing ART would ease the process significantly.

“I’ve scoured all of the Army leave pass regulation. I scoured the command policy. I scoured JTR [Joint Travel Regulations] as well as the Army Medical, Dental, and Veterinary policy’cause it’s all in one, and none of’em have any mention of artificial reproductive techniques. There’s no mention of elective versus nonelective procedures, nothing.”

#### Toll

Physical and mental fitness is a crucial element in the warrior ethos, and leaders expected participants to be disciplined, physically and mentally tough, and trained and proficient in their tasks and drills. But navigating inconsistent and fragmented care while balancing military and family life exacted a unique physical and emotional toll on participants.

##### Physical toll.

The physical effects of infertility treatments posed unique military considerations. Participants did not have the luxury of staying home during periods of discomfort or fatigue because of treatments. Physical fitness, weight standards, and mission readiness are key requirements, and several participants said they often felt torn between maintaining these standards and attempting to conceive.

“My physical fitness has suffered quite a bit in doing hormonal treatments, because either I’m really tired or exhausted or something just doesn’t feel right.”

“Increased bloating in the abdominal area was difficult to deal with when uniform inspections were coming up.”

##### Emotional toll.

Feelings of frustration, fear, loss, and confusion negatively impacted ADSMs. For self-preservation, participants isolated themselves from events that would trigger sadness. They directed anger toward perceived barriers or systems that contributed to the loss of hope. This was demonstrated in a military-specific fashion when participants experienced isolation because of deployment-related separation from spouses and partners, living in remote locations, and PCS because of military assignments.

“We’re overseas, the live-in, government housing is mandatory. We requested a release from housing because that’s where the daycare center is and the kiddy events. I said, ‘I can’t handle living there.’ He had to open up to his command and tell them what we were going through [ART] in order to request a release for us to not live in that type of environment. Which they denied, but—we didn’t wanna live in Coast Guard housing … there are kids everywhere.”

“The whole mental-health portion of this process is overlooked … Mental health already has stigma in the military as it is … Going through infertility … it’s very depressing for me. Now I’m struggling with infertility and depression. And it’s a double-whammy because both of those things … they’re just hard to talk about in the military.”

“Being here by myself for a year without my spouse has been extremely difficult, especially … that we’d have to go through IVF … It’s been tough on all of us because he sees that it upsets me, and I know that it upsets him … the family feeds off of it. The kids see the duress in the situation.”

### Moving Forward/ART Disengagement/Career Outcome

Some participants experienced successful births and were grateful to go through the ART process. Others disengaged from ART or reevaluated their family formation plans. Participants who remained actively engaged in care acknowledged they would face a time when they might choose to alter their path by adopting, fostering, or remaining childless. This endpoint involved a discussion about their military career. For some, military service did not align with their family goals and they chose to leave service.

“—he was in Charleston. I requested to go to Charleston, and they gave me orders to Georgia. So, I got off active duty. Moved to South Carolina.”

“I would tell people, ‘If you want a family, get out.’ You know, I would not encourage them to stay in …”

## DISCUSSION AND CONCLUSION

We sought to understand how ADSMs and their partners navigate the ART process within the MHS while managing a military career. Our participants endured unique challenges in decision-making regarding reproduction. ADSMs’ self-sacrifice and dedication to service conflicted with their desires for family and often superseded decisions for infertility care. When ADSMs chose to start a family and needed ART, DoD health care processes, command culture, and military policy varied widely and were critical facilitators and barriers to care. Overall, this resulted in fragmented and inconsistent care cycles, which contributed to increased emotional and physical stress and tension between career progression and family formation.

Barriers to ART access are epidemiological, financial, geographic, and socioeconomic, as well as related to patient and provider knowledge and government and health care policy.^8^ Many ADSMs serve during their childbearing years when deployments and mission requirements present a threat to fertility because of reproductive delay and partner separation.^6,9,10^ Women report electing to delay childbearing beyond their original plans for military readiness.^9^ Among women veterans, reporting a history of infertility has been associated with worse physical health-related quality of life and higher levels of mental health disorders than among civilians.^11–13^

As revealed in our study, female ADSMs have reported being denied ART because they were gay or unmarried; because of limited MTF capacity, treatment options, and TRICARE coverage; or via long waiting lists.^14^ A 2018 report found only 25 active duty reproductive endocrinologists who deploy overseas in support of military operations,^6^ which may further delay care. Overall, 47% of patients who receive ART care travel more than 50 miles for treatment,^15^ and half of the 700 ART cycles performed at Walter Reed National Military Medical Center were for patients who lived outside the Washington, DC metro area.^6^ Furthermore, ART is reported as a significant financial burden.^15^

We introduce new understandings of how command climate, career stage, policy, military physical requirements, and treatment availability near duty stations impact navigation of the ART process. We move beyond description and offer a theory of the infertility process that is grounded in the experience of our ADSM participants (Figs. 2 and 3). Our study’s findings are limited by the inclusion of few health care providers, same-sex couples, or single or enlisted members. Further research is needed to identify gaps in knowledge and access to ART based on military rank/rate, gender, occupation, and military branch.

Since high rates of infertility are related to military service and may result in increased risk for mental health concerns and reduced retention, we propose the following policy reforms: First, increase access to care by allowing ADSMs travel orders to the nearest MTF, offer the full range of ART at no cost at MTFs, and provide referrals for fertility preservation counseling to allow family planning around mission. Second, provide ADSMs, health care providers, and leaders with education on age-related fertility decline, the potential impact of delayed childbearing on future reproductive success, and fertility care options. Third, develop and make available a catalog of ART resources for ADSMs, including services provided by nonprofit organizations. Fourth, educate health care providers and military leaders regarding the mental and physical toll associated with infertility. Fifth, recognize that current policies amplify existing gender inequities and take steps to ensure equitable reproductive care for men and women through engagement with military leaders.
